# Freezing and water availability structure the evolutionary diversity of trees across the Americas

**DOI:** 10.1126/sciadv.aaz5373

**Published:** 2020-05-06

**Authors:** Ricardo A. Segovia, R. Toby Pennington, Tim R. Baker, Fernanda Coelho de Souza, Danilo M. Neves, Charles C. Davis, Juan J. Armesto, Ary T. Olivera-Filho, Kyle G. Dexter

**Affiliations:** 1 School of GeoSciences, University of Edinburgh, Edinburgh, UK.; 2 Instituto de Ecología y Biodiversidad, Santiago, Chile.; 3 Tropical Diversity Section, Royal Botanic Garden Edinburgh, Edinburgh, UK.; 4 Department of Geography, University of Exeter, Exeter, UK.; 5 School of Geography, University of Leeds, Leeds, UK.; 6 Departamento de Engenharia Florestal, Universidade de Brasília (UNB), Campus Universitário Darcy Ribeiro, Asa Norte, Brasília 70910-900, Brazil.; 7 Department of Botany, Federal University of Minas Gerais, Belo Horizonte, Brazil.; 8 Department of Organismic and Evolutionary Biology, Harvard University, Cambridge, MA, USA.; 9 Departamento de Ecología, Universidad Católica de Chile, Santiago, Chile.; 10 Facultad de Ciencias Naturales y Oceanográficas, Universidad de Concepción, Concepción, Chile.

## Abstract

The historical course of evolutionary diversification shapes the current distribution of biodiversity, but the main forces constraining diversification are still a subject of debate. We unveil the evolutionary structure of tree species assemblages across the Americas to assess whether an inability to move or an inability to evolve is the predominant constraint in plant diversification and biogeography. We find a fundamental divide in tree lineage composition between tropical and extratropical environments, defined by the absence versus presence of freezing temperatures. Within the Neotropics, we uncover a further evolutionary split between moist and dry forests. Our results demonstrate that American tree lineages tend to retain their ancestral environmental relationships and that phylogenetic niche conservatism is the primary force structuring the distribution of tree biodiversity. Our study establishes the pervasive importance of niche conservatism to community assembly even at intercontinental scales.

## Teaser

Freezing temperatures and water availability shape the distribution of evolutionary lineages of angiosperm trees in the Americas.

## INTRODUCTION

A central challenge in biogeography and macroevolution is to understand the primary forces that drove the diversification of life and the assemblage of ecological communities. Was diversification confined within continents and characterized by adaptation of lineages to different major environments (i.e., biome switching), or did lineages tend to disperse across great distances but retain their ancestral environmental niche (i.e., phylogenetic niche conservatism)? Classically, the attempts to define biogeographic regions based on shared plant and animal distributions lend support to the first hypothesis, that large-scale patterns may be explained by regionally confined evolutionary diversification, rather than long-distance dispersal (*1*–*3*). However, recent studies of the distribution of plant lineages at global scales have documented high levels of intercontinental dispersal [e.g., (*4*–*8*)] and revealed that lineages tend to retain their ancestral biomes when dispersing (*9*, *10*). These recent findings suggest that environmental associations of lineages may be the primary force organizing the course of diversification, but a key knowledge gap is in studies comparing the degree of evolutionary similarity among species assemblages at large geographic scales. Taking advantage of recent advances in the availability of broadscale biodiversity and genomic data and appropriate analytical methods (*11*), we unveil the evolutionary structure of tree assemblage diversity at an intercontinental scale.

With high mountain chains running north to south across latitudes and a mosaic of contrasting environments, the Americas represent a natural laboratory to investigate the evolutionary forces behind community assembly and the modern distribution of biodiversity. Here, we examine the phylogenetic composition of angiosperm tree assemblages across the Americas as a means to determine whether dispersal limitation or phylogenetic niche conservatism had a greater impact on the present-day evolutionary composition of tree assemblages. If lineages tend to retain their environmental niche as they diversify across space, then we would expect major evolutionary groups to be restricted to specific environmental regimes. This leads to the prediction that lineage composition of assemblages from extratropical regions in both hemispheres should be more similar to each other than to assemblages that occur in intervening tropical regions. In addition, we would predict that assemblages from dry tropical environments should show greater similarity in tree lineage composition to each other than to assemblages from moist environments with which they may be spatially contiguous (*12*). Alternatively, if diversification is spatially restricted and biome switching is common, then the major evolutionary grouping of assemblages should be segregated geographically. Thus, we would predict assemblages from South America (which was physically isolated through the Cenozoic) to constitute one group and assemblages from North and Central America to constitute another.

To test the relative importance of phylogenetic niche conservatism versus dispersal limitation, we analyzed data from ~10,000 tree assemblages with a new, temporally calibrated genus-level phylogeny that includes 1358 genera (~90% of tree genera sampled per assemblage). We assessed similarity in lineage composition among assemblages using clustering analyses and ordinations based on shared phylogenetic branch length. Next, we identified the indicator lineages for each major group in the clustering analysis and explored the geographic and environmental correlates of the distribution of the main evolutionary clusters. We further assessed the degree to which climatic variables versus geographic position could classify sites into different evolutionary groups. If climatic variables provide a better means of distinguishing groups than geographic variables, then this would support the idea that phylogenetic conservatism is more important than dispersal limitation in determining the distribution of evolutionary lineages, while the converse would hold if geographic variables perform better. Last, we estimated the unique evolutionary diversity (i.e., sum of phylogenetic branches of lineages restricted to individual groups) versus shared evolutionary diversity (i.e., sum of shared phylogenetic branches) across evolutionary groups (for details, see Materials and Methods section).

## RESULTS

**Fig. 1:**
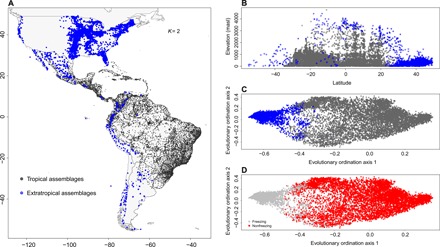
The geographic, evolutionary, and environmental relationships of the two principal evolutionary groups (from *K* = 2 clustering analysis). (**A**) Geographic distribution of angiosperm tree assemblages and their affiliation with either the tropical (*n* = 7145) or extratropical (*n* = 2792) evolutionary group. (**B**) Distribution of assemblages over elevation and latitude, showing that the extratropical group is largely restricted to high elevations at low latitudes. (**C** and **D**) Distribution of assemblages over the first two axes of an ordination based on evolutionary composition with assemblages in (C) colored according to group affiliation and in (D) as to whether or not they experience freezing temperatures in a regular year [from (*50*)]. masl, meters above sea level.

**Fig. 2:**
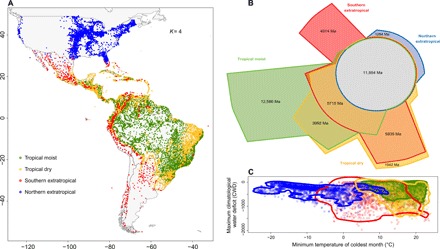
The geographic, evolutionary, and environmental relationships among four evolutionary groups (from *K* = 4 clustering analysis). (**A**) Geographic distribution of angiosperm tree assemblages and their affiliation with one of the four evolutionary groups. (**B**) Euler diagram representing the amount of evolutionary history, quantified as phylogenetic diversity (PD) (in millions of years), restricted to each cluster versus that shared between clusters. (**C**) Distribution of assemblages over extremes of temperature (minimum temperature of coldest month) and water availability [maximum climatological water deficit (CWD)]. Lines represent the 95th quantile of the density of points for each group.

**Fig. 3:**
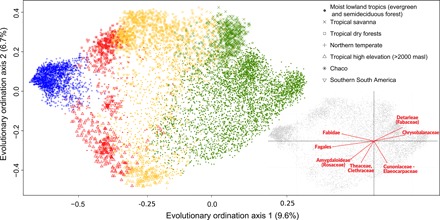
Phylogenetic ordination of tree assemblages based on their evolutionary lineage composition. Colors in the main plot represent the groups from *K* = 4 clustering analyses and the different symbols represent major vegetation formations. The subset plot shows the clades most strongly associated with the first two axes of the evolutionary ordination.

We show that the evolutionary lineage composition of American tree assemblages is structured primarily by phylogenetic niche conservatism. The two principal groups (*K* = 2) have a tropics-extratropics structure (Fig. 1). The extratropical group is not geographically restricted, but includes temperate tree assemblages from North America and southern South America, connected by a high-elevation corridor in low latitudes (Fig. 1, A and B). The tropics-extratropics structure of tree evolutionary diversity shows a strong correspondence (97% match, fig. S1) with the absence versus occurrence of freezing temperatures within a typical year (see Fig. 1, C and D). We observe that most evolutionary diversity, measured as summed phylogenetic branch length, occurs within the tropics, but that there is unique evolutionary diversity restricted to the extratropics (~10% of the total; Fig. 2B and fig. S3A). Ordination and indicator clade analyses revealed that the tropics-extratropics segregation is associated with the distribution of specific clades, such as the Fagales, which includes the oaks (*Quercus*), beeches (*Fagus*), coihues (*Nothofagus*), and their relatives (Fig. 3 and tables S1 and S2).

On the basis of two different analyses (Elbow and Silhouette methods; see Materials and Methods for discussion of selecting optimal *K*), clusters of *K* = 3 and *K* = 4 groups are also supported as additional informative splits (fig. S2), and each of their major groups capture substantial unique evolutionary diversity (Fig. 2B, fig. S3, and table S2). In *K* = 3, the main extratropical cluster grouped assemblages from North America and extreme southern South America, while the remaining assemblages from temperate southern South America and the Andean tropics grouped with assemblages from the arid or semiarid tropics and subtropics (fig. S4). The third group was formed by the moist tropics (fig. S4). For *K* = 4, the extratropics were split into a largely temperate North American group and a second group that joins subtropical sites in South and Central America with southern temperate forests and high elevation sites in the Andes (Fig. 2A). In the tropics, there is one group uniting assemblages found in ever-moist and warm conditions, and a second group of assemblages that extend into drier and subtropical areas (Fig. 2C and fig. S5A), including most tropical dry forest assemblages (Fig. 2A and Table S3). We refer to the four groups of assemblages in *K* = 4 as the northern extratropical, southern extratropical, tropical moist, and tropical dry groups.

Focusing on the *K* = 4 analyses, we found that climatic variables perform markedly better than geographic variables in classifying individual assemblages into evolutionary groups, supporting the preeminence of phylogenetic niche conservatism as opposed to dispersal limitation in structuring the distribution of biodiversity in tree assemblages. A simple climatic model with mean annual precipitation (MAP), mean annual temperature (MAT), maximum climatological water deficit (CWD), and temperature seasonality (TS) succeeded in classifying 86.4% of assemblages, on average, into the correct evolutionary group. A simple geographic model, that South American assemblages should fall into a separate group from North and Central American assemblages, and with latitude and longitude as input variables, classified 76.0% of assemblages correctly on average. Adding latitude and longitude may even overemphasize the importance of geography given that latitude and longitude are correlated with climatic variation. In the climatic classification for *K* = 4 groups, temperature variables surpass precipitation variables as the most important classificatory variables [mean decrease in Gini index when excluded (*13*); for TS, 2728; MAT, 1565; MAP, 1064; and CWD, 936]. When focusing only on the tropics, these climatic variables correctly classify sites 83.6% of the time, with the most important variable being CWD (mean decrease in a Gini index of 792), followed by MAT (722), TS (643), and MAP (642). When focusing only on the extratropics, these climatic variables correctly classify sites 98.4% of the time. TS was by far the most important variable (mean decrease in a Gini index of 719), which is in line with previous research showing that Southern Hemisphere temperate areas are less seasonal than Northern Hemisphere temperate areas (*14*, *15*). TS was followed in importance by CWD (157), MAP (92), and MAT (75). Analyses with generalized linear models suggest that MAP is the most important climatic variable to distinguish assemblages in the tropical moist from tropical dry groups and that TS is the most important to distinguish the two extratropical groups (fig. S6).

## DISCUSSION

Our results demonstrate that the tropics-extratropics evolutionary structure of tree diversity is principally associated with the environmental threshold of the presence versus absence of freezing temperatures (Fig. 1, A and B, and fig. S1). This pattern is consistent with evidence documenting that only angiosperm lineages that were able to evolve traits to avoid freezing-induced embolism radiated into high latitudes (*16*). In addition, we determined that a unique, sizeable portion of the total evolutionary diversity of angiosperm trees is restricted to extratropical assemblages, as the fossil record corroborates (*17*, *18*). Collectively, this evidence suggests that the phylogenetic conservatism of lineages from the extratropics has a major relevance for the diversification of angiosperm trees in the Americas. Kerkhoff *et al.* (*19*) estimated that in the extratropical region (defined by them as areas north of 23°N and south of 23°S), angiosperm lineages produced extratropical descendants at least 90% of the time. Considering that some areas subjected to regular freezing at high elevations in equatorial latitudes may be better classified as extratropical, as demonstrated here by our results (Fig. 1), extratropical phylogenetic conservatism could even be greater than found by Kerkhoff *et al.* (*19*).

We suggest that extratropical conservatism has a major importance in the biogeography of the Americas. The relatively recent uplift of the Andes would have created novel environments, with regular freezing temperatures, at low latitudes. Freezing temperatures would have filtered dispersal into this new habitat, allowing extratropical lineages to move from both north and south to equatorial latitudes (*20*, *21*), but constraining the immigration of lineages from lowland, frost-free environments. Fossil pollen demonstrates the arrival in the northern Andes of tree genera from temperate forests in the Northern Hemisphere, including *Juglans* (Juglandaceae), *Alnus* (Betulaceae), and *Quercus* (Fagaceae), at about 2.2 million years (Ma), 1.0 Ma, and 300,000 years, respectively, and the arrival of southern genera, including *Weinmannia* (Cunoniaceae) and *Drymis* (Winteraceae), during the late Pliocene and Pleistocene (1.5–3.2 Ma) (*20*, *22*). Likewise, phylogenetic evidence shows recent diversification in the Andes of lineages that seem to have originated in the extratropics, including *Lupinus* (Fabaceae) (*23*), Adoxaceae/Valerianceae (*24*, *25*), and *Gunnera* (Gunneraceae) (*26*).

Our results also point to a moist versus dry evolutionary divide within the Neotropics. Tropical moist group assemblages hold the greatest amount of evolutionary diversity, both overall and unique to them, despite occupying the most restricted extent of climatic space of any of the *K* = 4 groups (Fig. 2, B and C). Tropical dry group assemblages, in contrast, extend across a broader climatic space, but hold less evolutionary diversity overall (Fig. 2, B and C). This asymmetry in the accumulation of diversity may reflect phylogenetic conservatism for a putatively moist and hot ancestral angiosperm niche (*27*), or could result from a favorable environment in tropical moist regions that can be occupied by any angiosperm lineage, even those that also occur in cooler or drier conditions (*28*, *29*). Regardless, the similarity in the lineage composition of the extensive but discontinuously distributed tropical dry forests (*12*) indicates their separate evolutionary history. Tropical dry forests have been described as dispersal limited (e.g., *12*), but this refers to the ability of constituent taxa to persist locally over evolutionary time scales, thereby inhibiting immigration. However, even a low rate of dispersal and immigration among American tropical dry forest regions suffices to maintain floristic cohesion. Such evolutionary isolation of the dry forest flora has previously been suggested by studies in Leguminosae (*12*, *30*), and is shown here to be evident at the evolutionary scale of all angiosperm tree species.

Our results also help to clarify the contentious evolutionary status of savanna and Chaco regions in the Neotropics. We find that the southern savannas (the Cerrado region of Brazil) are more evolutionary related to tropical moist forests than dry forests (Fig. 2A, and fig. S4), as previously suggested for specific clades (*30*, *31*). However, northern tropical savannas (i.e., Llanos of Venezuela and Colombia and those in Central America) are split in their evolutionary linkages between the tropical moist and tropical dry groups (Fig. 3 and table S3). This may reflect the distinct ecology of many northern savannas [e.g., the Llanos are hydrological savannas (*32*)] and suggest a divergent evolutionary history for northern and southern savannas. Our results may also help to resolve the debates around the evolutionary affinities of the Chaco [e.g., (*33*, *34*)], by showing that this geographically defined region houses a mix of extratropical and tropical lineages (Fig. 2).

More broadly, our analyses consistently point to evolutionary links between assemblages in seasonally dry and seasonally cold areas (Fig. 2 and fig. S4). For example, when we consider *K* = 3 evolutionary groups, a single “dry and cool” group coalesces, including southern South American extratropics, seasonally tropical dry forests, and Mexican pine-oak forests, with the other two groups being the tropical moist forest group and a largely northern, extratropical group (fig. S4). Along the same lines, the southern extratropical group from the *K* = 4 clustering also includes subtropical forests in arid and semiarid regions of Chile, Mexico, and elsewhere (Fig. 2), while the tropical dry group includes tree assemblages occurring in cool areas at high elevation, largely in the southern Atlantic Forest of Brazil (Fig. 2). When we consider *K* = 5 evolutionary groups, these cool sites, which are also moister than the rest of the tropical dry group, split off to form a fifth group that also takes in sites at higher elevation in the Andes, the Guianan Highlands, Central America, and the Caribbean (fig. S5).

We show that the evolutionary composition of tree assemblages in the Americas is determined primarily by the presence versus absence of freezing temperatures, dividing tropical from extratropical regions. Within the tropics, we find further evolutionary subdivision among assemblages experiencing moist versus seasonally dry conditions. These findings demonstrate that phylogenetic niche conservatism is the primary force organizing the diversification, community assembly, and, therefore, the biogeography of angiosperm trees. Tree species that can inhabit areas experiencing freezing temperatures and/or environments subjected to seasonal water stress belong to a restricted set of phylogenetic lineages, which gives a unique evolutionary identity to extratropical forests and tropical dry forests in the Americas. While our study is restricted to New World trees, we suggest that plant biodiversity globally may be evolutionarily structured following a tropics-extratropics pattern, while diversity within the tropics may be structured primarily around a moist-dry pattern. These findings advocate strongly for integrating the concepts of extratropical conservatism and tropical-dry conservatism into our understanding of global macroevolutionary trends and biogeographic patterns.

## MATERIALS AND METHODS

### Tree assemblage dataset

Our tree assemblage dataset was derived by combining the NeoTropTree (NTT) database (*35*) with selected plots from the Forest Inventory and Analysis (FIA) Program of the U.S. Forest Service (*36*), accessed on 17 July 2018 via the BIEN package (*37*). Sites in the NTT database are defined by a single vegetation type within a circular area of 5-km radius and contain records of tree and tree-like species, i.e., freestanding plants with stems that can reach over 3 m in height [see www.neotroptree.info and (*38*) for details]. Each FIA plot samples trees that are ≥12.7-cm diameter at breast height in four subplots (each being 168.3 m^2^) that are 36.6 m apart. We aggregated plots from the FIA dataset within 10-km-diameter areas, to parallel the spatial structure of the NTT database. We excluded any sites that had less than five angiosperm genera, as preliminary analyses suggested that these sites lacked sufficient information to be confidently placed in evolutionary ordinations and clustering described below. Therefore, the FIA dataset was reduced considerably, and some regions with a low diversity of angiosperms have no samples in our study. This procedure produced a total dataset of 9937 tree assemblages distributed across major environmental and geographic gradients in the Americas.

### Genus-level phylogenetic tree

We obtained sequences of the *rbc*L and *mat*K plastid gene for 1358 angiosperm tree genera, from GenBank (www.ncbi.nlm.nih.gov/genbank/), building on previous large-scale phylogenetic efforts for angiosperm trees in the Neotropics (*39*, *40*). Sequences were aligned using the MAFFT software (*41*). “Ragged ends” of sequences that were missing data for most genera were manually deleted from the alignment.

We estimated a maximum likelihood phylogeny for the genera in the RAxML v8.0.0 software (*42*) on the CIPRES web server (www.phylo.org). We constrained order-level relationships in the tree, following the phylogeny in Gastauer *et al.* (*43*), which is based on the topology proposed by the Angiosperm Phylogeny Group IV. We concatenated the two chloroplast markers following a general time reversible + gamma model of sequence evolution. We included sequences of *Nymphaea alba* (Nymphaeaceae) as an outgroup. We used a maximum likelihood bootstrap analysis to assess support for relationships in the phylogeny. Most deeper relationships in the phylogeny had high support values (>70 bootstrap support), which is expected given that ordinal relationships were fixed. More recent nodes in the phylogeny had lower support with the relationships of genera within families having mean bootstrap support values of ~60. However, we confirmed that relationships of families within orders and genera within families generally matched those in more detailed phylogenetic analyses (with more variable genetic markers), specifically those studies listed in table S4. The low support values are likely attributable to the relatively low variability of the *mat*K and *rbc*L markers within angiosperm families.

We temporally calibrated the maximum likelihood phylogeny using the software treePL (*44*). We implemented age constraints for 320 internal nodes [family level or higher, from (*45*)] and for 123 genera stem nodes (based on ages from a literature survey; table S4). The rate smoothing parameter (lambda) was set to 10 based on a cross-validation procedure. The final dated phylogeny can be found in the Supplementary Materials.

### Phylogenetic distance analysis and clustering

We used the one complement of the Phylosor index (i.e., 1 − Phylo-Sorensen) to build a matrix of phylogenetic dissimilarities between plots based on genera presence-absence data. The Phylosor index sums the total branch length of shared clades between sites (*46*) relative to the sum of branch lengths of both sites

Complement of Phylo−Sorensen ij=1−BLij/0.5*(BLi+BLj)

where BL*ij* is the sum of shared phylogenetic branch length between sites *i* and *j*, and BL*i* and BL*j* are the sum of branch length of phylogenies comprising solely genera within sites *i* and *j*, respectively. Thus, if all branches are shared between two plots, then the dissimilarity measure takes on a value of 0. If no branches are shared between plots (i.e., the plots comprise two reciprocally monophyletic clades), then the dissimilarity measure will take on a value of 1. This metric was estimated using the phylosor.query() function in the PhyloMeasures (*47*) package for R. Analyses with the one complement of the Unifrac phylogenetic similarity measure gave highly similar results and are not presented here.

We used *K*-means clustering to explore the main groups, in terms of (dis)similarity in the tree assemblage dataset, according to the Phylosor dissimilarity measures. Preliminary analyses using hierarchical clustering approaches did not produce coherent groupings. The *K*-means clustering algorithm requires the number of groups/clusters (*K*) to be specified in advance. To estimate the best value for *K*, the optimal number of clusters to parsimoniously explain the variance in the dataset, we used the Elbow method and an approach based on the average Silhouette width (fig. S2). The Elbow method assesses how the total within-cluster sum of squares (TSS) changes as a function of the number of clusters. Each additional cluster lowers the TSS, and the elbow of the curve is formed when adding another cluster fails to lower the TSS substantially compared to previous increases in cluster number. On the other hand, the Silhouette width analysis determines how well each assemblage fits within its assigned evolutionary group/cluster, with higher values indicating that the site is closer compositionally to the “median” composition (i.e., centroid) of its assigned group relative to its proximity to the “median” composition of the other groups. The higher the average silhouette width across all assemblages, the better the clustering. The Elbow analyses suggest anything from *K* = 3 to *K* = 5 to be the best clustering, and the Silhouette width analysis point to *K* = 2 to be the best clustering. On the basis of these results, we selected *K* = 2 (Fig. 1), *K* = 3 (fig. S4), *K* = 4 (Fig. 2), and *K* = 5 (fig. S5) for further analysis and interpretation. No geographic or environmental data were used to inform the clustering analyses (*48*). The *K*-means clustering was carried out with the kmeans() function in base R (R Core Development Team, 2016).

In addition, we performed an evolutionary ordination of tree assemblages based on their phylogenetic lineage composition, following protocols developed by Pavoine (*49*). We specifically used an evolutionary principal components analysis, implemented with the evopca() function in the “adiv” package (*49*), with a Hellinger transformation of the genus by site matrix, as this is a powerful approach to detect phylogenetic patterns along gradients, while also allowing positioning of sites and clades in an ordination space (*11*). The first two axes explained 9.6 and 6.7% of the variation in the data, with subsequent axes each explaining <5.5%.

### Correspondence between clustering results and environmental variables

We tested the correlation between our *K* = 2 clustering result and eight different delimitations of the tropics, as per Feeley and Stroud (*50*). These delimitations were as follows: (C1) all areas between 23.4°S and 23.4°N; (C2) all areas with a net positive energy balance; (C3) all areas where MAT does not co-vary with latitude; (C4) all areas where temperatures do not go below freezing in a typical year; (C5) all areas where the mean monthly temperature is never less than 18°C; (C6) all areas where the mean annual “biotemperature” ≥24°C; (C7) all areas where the annual range of temperature is less than the average daily temperature range; and (C8) all areas where precipitation seasonality exceeds TS. We calculated the correspondence between our binary clustering (i.e., tropical versus extratropical) and each of these delimitations as the proportion of sites where the delimitations matched.

To assess whether the *K* = 4 clustering is mainly influenced by climate or by geography, we determined the proportion of assemblages that can be correctly categorized into their evolutionary group by environmental variables versus spatial variables, using a random forest classification tree approach (*13*). The explanatory variables for the environmental model were MAT, MAP, and TS from the Worldclim dataset (*51*) and maximum CWD from Chave *et al.* (*52*). For the geographic model, we used a basic division between South America versus North and Central America together, as this reflects the historic geographic isolation of South America. We also included latitude and longitude as explanatory variables in this basic geographic model. We excluded sites in the Caribbean from both models as it was not certain how to group them in the geographic model. Even including them would not have changed the results substantially as they only comprise 2.4% of sites in our total assemblage database. These analyses were implemented with the randomForest() function in the “randomForest” package (*13*).

To explore the best climatic variable to explain the divisions between groups within the tropics and the extratropics, we used a mixed model with a binomial response (tropical dry versus tropical moist for the tropics and extratropical north versus extratropical south for the extratropics). To account for spatial autocorrelation, we grouped assemblages in 1° × 1° grid cells and incorporated the many-level grid cell factor as a random effect. We implemented the mixed model with the function glmer() from the lme4 package (*53*). To determine the best climatic variable, we compared the models based on the Akaike information criterion (AIC). As candidate variables, we focused on the same variables as in the random forest analysis, MAT, MAP, TS, and CWD.

### Shared versus unique PD

As the Phylosor estimation of evolutionary (dis)similarity cannot distinguish variation associated to differences in total PD, or phylogenetic richness, versus variation associated to phylogenetic turnover per se, we measured the shared and unique PD associated with each group for the *K* = 2, *K* = 3, and *K* = 4 clustering analyses. First, we estimated the association of genera with each group by an indicator species analysis following de Caceres *et al.* (*54*). Specifically, we used the multipatt() function in the R Package indicspecies (*55*) to allow genera to be associated with more than one group (when *K* > 2). The output of the multipatt function includes the stat index, which is a function of the specificity (the probability that a surveyed site belongs to the target site group given the fact that the genus has been found) and fidelity (the probability of finding the genus in sites belonging to the given site group). We constructed pruned phylogenies excluding those genera with specificity greater than 0.6 for a group, or combination of groups, to estimate the total PD found in each group or combination of groups without their specific indicators. Then, we subtracted these totals from the entire total for the complete, unpruned phylogeny to determine the amount of phylogenetic diversity restricted to each group or combination of groups. Last, we estimated the PD shared across all groups as that which was not restricted to any particular group or any combination of groups. We fit these different PD totals as areas in a Euler diagram with the euler() function in the “eulerr” package (*56*) for the *K* = 2 and *K* = 3 clustering and with the Venn() fuction in the “venn” package (*57*) for the *K* = 4 clustering.

### Indicator lineages for clusters

To further characterize the composition of the evolutionary groups, we conducted an indicator analysis to determine the evolutionary clades most strongly associated with each group. We created a site × node matrix, which consists of a presence/absence matrix for each internal node in the phylogeny and ran an indicator analysis for the nodes. We selected the highest-level, independent (i.e., non-nested) nodes with the highest stat values to present in tables S1 and S2. The indicator node analysis was carried out with function multipatt() in the R Package indicspecies (*55*).

## Supplementary Material

aaz5373_SM.pdf

## SUPPLEMENTARY MATERIALS

Supplementary material for this article is available at http://advances.sciencemag.org/cgi/content/full/6/19/eaaz5373/DC1

## Appendix

View/request a protocol for this paper from *Bio-protocol*.
