# Supplementary material for: Freezing and water availability structure the evolutionary diversity of trees across the Americas
Source: Sci Adv. 2020 May 6;6(19):eaaz5373. doi: 10.1126/sciadv.aaz5373 (PMC7202884; doi:10.1126/sciadv.aaz5373)
Supplement: aaz5373_SM.pdf [file aaz5373_SM.pdf]

Science Advances
AAAS

advances.sciencemag.org/cgi/content/full/6/19/eaaz5373/DC1

Supplementary Materials for

Freezing and water availability structure the evolutionary diversity of trees across the Americas

Ricardo A. Segovia*, R. Toby Pennington, Tim R. Baker, Fernanda Coelho de Souza, Danilo M. Neves, Charles C. Davis, Juan J. Armesto, Ary T. Olivera-Filho, Kyle G. Dexter

*Corresponding author. Email: ricardo.segovia@ed.ac.uk

Published 6 May 2020, *Sci. Adv.* **6**, eaaz5373 (2020)
DOI: 10.1126/sciadv.aaz5373

This PDF file includes:

Tables S1 to S4
Figs. S1 to S6
References

Table S1 Indicator clades for *K*=2 groups. Specificity, fidelity and indicator statistic (stat) of the top internal nodes with the highest indicator statistic for these groups. Clades names are based on their taxonomic composition.

Cluster	Clades	*Specificity*	*Fidelity*	stat
Tropical	Gentianales	0.9419787	0.9885234	0.965
	Fabaceae, tribe Mimoseae	0.9339992	0.9680896	0.951
	Fabaceae, subfam. Caesalpinioideae	0.9366716	0.9588523	0.948
	Euphorbiaceae and Peraceae	0.9639994	0.9303009	0.947
	Putranjivaceae, Phyllanthaceae, Picrodendraceae and Ochnaceae	0.9792854	0.9143457	0.946
Extratropical	Fagales	0.886934	0.85745	0.872
	Fagales *plus* Cucurbitales	0.875902	0.85745	0.867
	Ulmaceae	0.987098	0.62106	0.783
	Sapindaceae, subfam. Hippocastanoideae	0.943887	0.612106	0.76

Table S2 Indicator clades for *K*=4 groups. Specificity, fidelity and indicator statistic (*stat*) of the top internal nodes with the highest indicator statistic for these groups. Clade names are based on their taxonomic composition.

Cluster	Clades	*Specificity*	*Fidelity*	stat
Tropical Moist	*Xylopia*, *Fusaea* and *Duguetia* (Annonaceae)	0.849969	0.700198	0.771
	Couepia and *Hirtella* (Chrysobalanaceae)	0.757572	0.711399	0.734
	Burseraceae, tribes Protieae and Canarieae	0.794147	0.678014	0.734
	Ochnaceae	0.746723	0.706128	0.726
	Myristicaceae	0.851393	0.591917	0.71
	Calophyllaceae	0.7516	0.668351	0.709
Tropical Dry	Bignoniaceae, tribes Bignonieae and Tecomeae	0.778906	0.117489	0.303
	Lasiocarpus and *Ptilochaeta* (Malpighiaceae)	0.853331	0.072646	0.249
	Cactaceae, tribe Trichocereeae	0.864368	0.047982	0.204
Southern Extratropical	*Prosopis*, *Piptadeniopsis* (Fabaceae, tribe Mimoseae)	0.733744	0.402597	0.544
	Fabaceae, tribe Caesalpinieae	0.670481	0.301587	0.45
	Vallea and *Aristotelia* (Elaeocarpaceae)	0.854485	0.223665	0.437
	Cactaceae, tribes Pachycereeae and Notocactea	0.732889	0.248196	0.426
	Scrophulariaceae	0.597146	0.30303	0.425
Northern Extratropical	Sapindaceae, subfam. Hippocastanoideae	0.83986	0.69118	0.762
	Ulmaceae	0.75668	0.70459	0.73
	Oleaceae, tribe Oleeae	0.80497	0.62332	0.708
	Juglandaceae	0.76212	0.53677	0.64

Table S3. Affiliation of principal vegetation formations in the tropics with the two main tropical groups from the K=4 clustering analysis. Vegetation formations were taken from the NeoTropTree dataset, which categorises formations first based on physiognomy (savanna vs. forest) and then segregates the forests based on phenology. Following (*38*) and (*58*), we consider deciduous tropical forests to represent the tropical dry forest biome, while semideciduous forests are more related floristically to the tropical moist forest biome. Semideciduous forests share many tree species with evergreen forests and relatively few with more fully deciduous forests (*38*,*58*). We further divided the savannas based on geography, as our analyses showed evident differences in group affiliation between savannas in the Cerrado Domain of Brazil versus those further north (i.e. Llanos of Venezuela and Colombia and those in Central America).

	Tropical dry	Tropical moist
Evergreen Forests	15% (501)	85% (2948)
Semideciduous Forests	10% (167)	90% (1530)
Deciduous Forests	75% (868)	25% (285)
Southern Savannas (Cerrado)	8% (56)	92% (657)
Northern Savannas	54% (65)	46% (56)

Table S4. Stem ages for genera nodes. Ages used to calibrate the phylogenetic tree, and the reference of their source. References (*59*-*92*).

	Genus	stem age (Myr)	Reference
1	*Acer*	60	*(81)*
2	*Acioa*	19.1	*(64)*
3	*Aesculus*	65	*(72)*
4	*Anaxagorea*	90.44	*(62)*
5	*Andira*	17.51	*(62)*
6	*Antiaris*	34	*(71)*
7	*Aphananthe*	71.5	*(97)*
8	*Aphanocalyx*	46	*(65)*
9	*Artocarpus*	51	*(83)*
10	*Atuna*	20.5	*(64)*
11	*Avicennia*	70.09	*(93)*
12	*Bagassa*	67	*(71)*
13	*Bocageopsis*	5.98	*(62)*
14	*Brosimum*	48	*(62)*
15	*Caesalpinia*	48.3	*(65)*
16	*Carapa*	29.5	*(62)*
17	*Cassia*	45	*(65)*
18	*Castilla*	22	*(62)*
19	*Cecropia*	44	*(62)*
20	*Cedrela ///toona*	48.4	*(79)*
21	*Cedrelopsis*	18.94	*(61)*
22	*Centroplacus*	69	*(66)*
23	*Cercis*	47.3	*(65)*
24	*Chrysobalanus*	24.2	*(64)*
25	*Cissus*	67.99	*(84)*
26	*Clarisia*	70	*(71)*
27	*Coceveiba*	72	*(62)*
28	*Cornus*	74.03	*(96)*
29	*Couepia*	21.6	*(64)*
30	*Crudia*	45	*(65)*
31	*Cylicomorpha*	35.5	*(60)*
32	*Cynometra*	12.93	*(62)*
33	*Dacryodes*	38	*(62)*
34	*Dactyladenia*	15.9	*(64)*
35	*Dactylocladus*	39	*(78)*
36	*Dialium*	10.9	*(62)*
37	*Dicymbe*	12	*(62)*
38	*Diplotropis*	20.27	*(62)*
39	*Dipterix*	26.44	*(62)*
40	*Dipterocarpus*	47.7	*(73)*
41	*Drimys*	56.76	*(92)*
42	*Dryobalanops*	43.3	*(73)*
43	*Duguetia*	30.64	*(62)*
44	*Dycorynia*	10.9	*(62)*
45	*Embothrium*	39.3	*(85)*
46	*Eperua*	12.32	*(62)*
47	*Ficus*	58	*(71)*
48	*Froesia*	39.4	*(86)*
49	*Fusaea*	30.64	*(62)*
50	*Glochidion*	31.51	*(94)*
51	*Glycosmis*	32.54	*(61)*
52	*Guarea*	14.8	*(62)*
53	*Guatteria*	55.83	*(62)*
54	*Gyrocarpus*	72	*(76)*
55	*Hakea*	12.8	*(75)*

Genus	stem age (Myr)	Reference
56 *Harrisoinia*	57.99	(61)
57 *Helicostylis*	28	(62)
58 *Hennecartia*	15.58	(82)
59 *Hernandia*	76	(76)
60 *Hevea*	85	(62)
61 *Hopea*	21.6	(73)
62 *Hymenaea*	24	(65)
63 *Inga*	10	(62)
64 *Ipomoea*	34.97	(70)
65 *Iryanthera*	19	(62)
66 *Jacaratia*	27.5	(60)
67 *Lacunaria*	20.3	(86)
68 *Lomatia*	70.8	(77)
69 *Lonchocarpus*	15.07	(62)
70 *Lonicera*	43.37	(63)
71 *Maclura*	85	(71)
72 *Macrolobium*	32	(62)
73 *Magnistipula*	19	(64)
74 *Malmea*	19.99	(62)
75 *Manilkara*	32	(59)
76 *Maranthes*	20.5	(64)
77 *Meliosma*	67.34	(98)
78 *Mimusops*	35	(59)
79 *Mouriri*	90	(80)
80 *Myrtae tribe*	58.96	(95)
81 *Neocarya*	25.6	(64)
82 *Ormosia*	40.62	(62)
83 *Otoba*	17	(62)
84 *Parashora*	22.9	(73)
85 *Parkia*	45.5	(62)
86 *Peltogyne*	28.8	(62)
87 *Persea*	55.3	(74)
88 *Peumus*	55.66	(82)
89 *Poecilanthe*	40.99	(62)
90 *Poulsenia*	22	(62)
91 *Pourouma*	44	(62)
92 *Pradosia*	47.5	(90)
93 *Prosopis SA*	28.96	(67)
94 *Protium*	52.5	(62)
95 *Prunus*	60.7	(68)
96 *Pseudolmedia*	36	(62)
97 *Pseudowintera*	45.18	(92)
98 *Pseudoxandra*	15.09	(62)
99 *Pterocarpus*	16.66	(62)
100 *Quiina*	29	(86)
101 *Rhododendron*	58	(87)
102 *Richea*	22.31	(87)
103 *Sambucus*	45.49	(63)
104 *Sideroxylon*	74	(88)
105 *Slonaea*	79	(69)
106 *Sorocea*	59	(62)
107 *Spathelia*	19.21	(61)
108 *Swartzia*	45.96	(62)
109 *Tachigali*	4.65	(62)
110 *Tasmania*	69.98	(92)
111 *Tepualia*	24.9	(91)
112 *Unonopsis*	7.94	(62)
113 *Urophyllum*	27.1	(89)
114 *Vallea*	48	(69)
115 *Vateria*	15.4	(73)
116 *Vatica*	18.3	(73)
117 *Viburum*	71.18	(63)
118 *Virola*	17	(62)
119 *Vitellariopsis*	26	(59)
120 *Vouacapoua*	48.69	(62)
121 *Xylopia*	49.98	(62)
122 *Zygia*	17.82	(62)

Fig. S1. Match between tropics vs. extratropics groups from K=2 clustering and eight delimitations of the tropics following Feeley & Stroud [2018] ***(50)*****:** C1) all areas that occur between 23.4°S and 23.4°N; C2) all areas with a net positive energy balance; C3) all areas where mean annual temperature does not vary with latitude; C4) all areas where temperatures do not go below freezing in a typical year; C5) all areas where the mean monthly temperature is never less than 18°C; C6) all areas where the mean annual "biotemperature" ≥ 24°C; C7) all areas where the annual range of temperature is less than the average daily temperature range; C8) all areas where precipitation seasonality exceeds temperature seasonality.

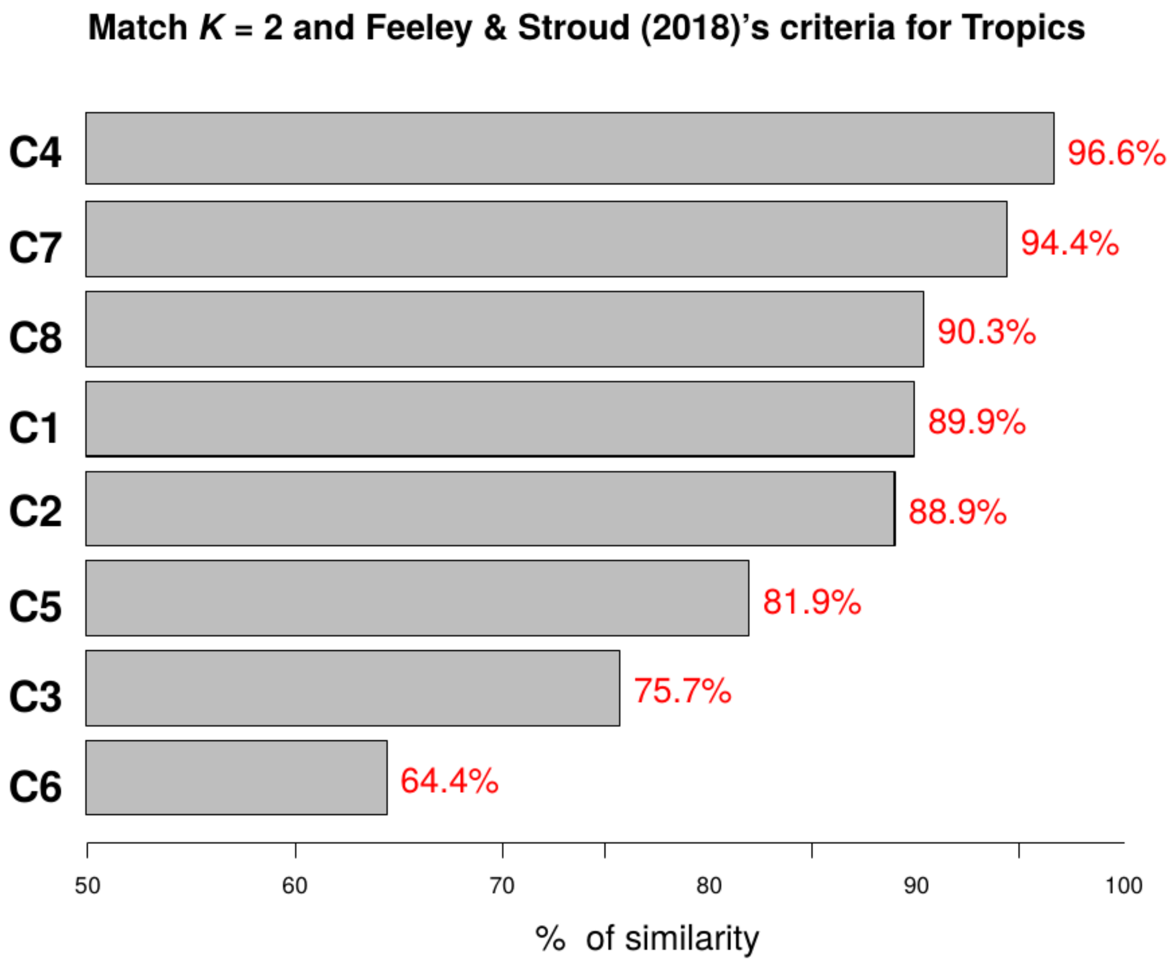

Fig. S2. Selection of number of clusters. A) Elbow criterion, explained variance from clustering as a function of number of groups; B) Silhouette criterion, average silhouette width for each site as a function of number of groups.

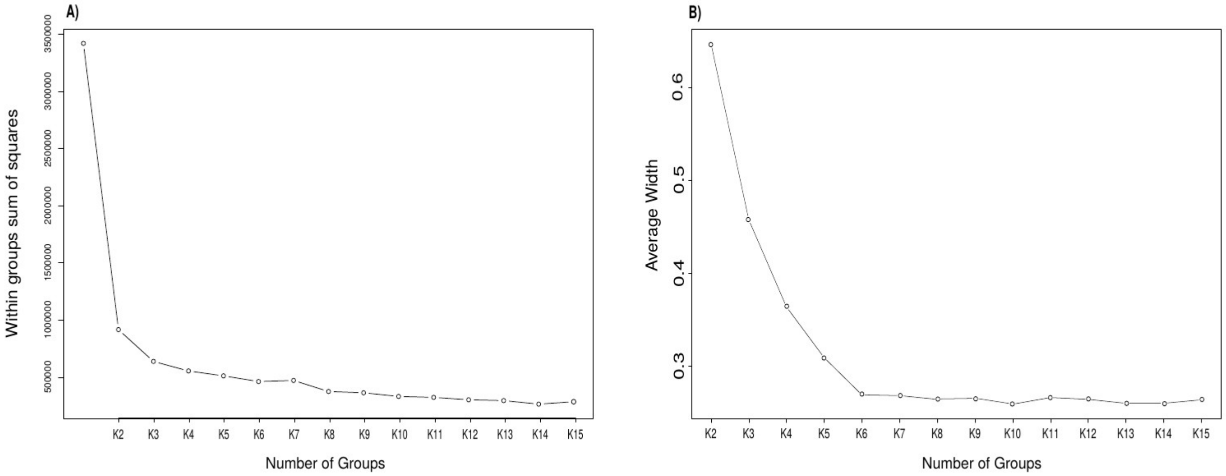

Fig. S3 Shared versus unique Phylogenetic Diversity for *K*=2 and *K*=3 clustering analyses. Euler Diagrams showing the amount of unique phylogenetic diversity in each cluster and the phylogenetic diversity shared between clusters (in millions of years). A) *K*=2 clustering and B) *K*=3 clustering.

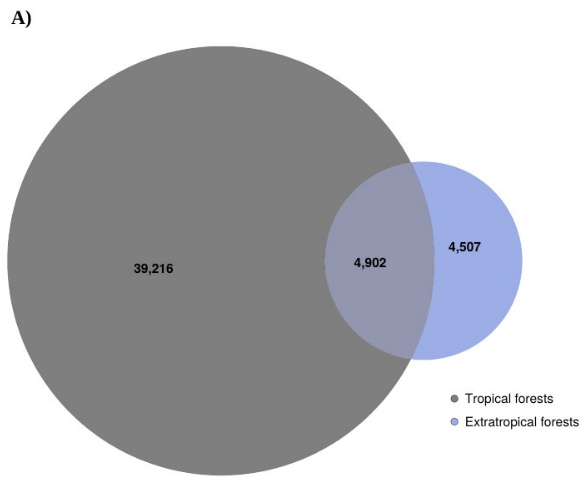

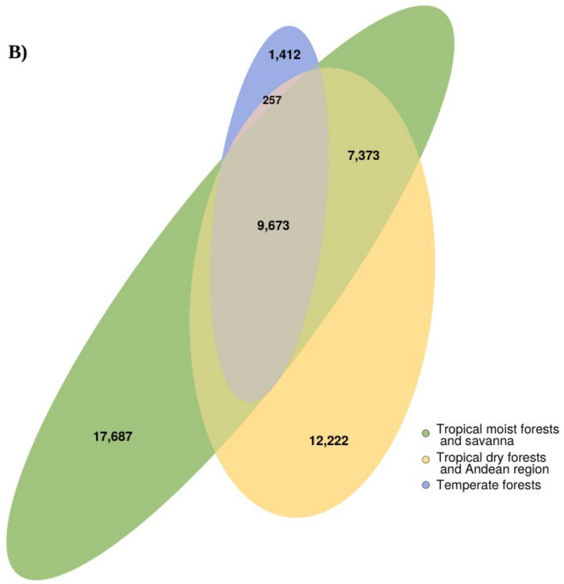

Fig. S4. Clustering *K* = 3. A) Location of the 9937 angiosperm tree assemblages in three evolutionary groups; B) Ordination of tree assemblages based on evolutionary lineage composition; C) Maximum Climatological Water Deficit (CWD) versus minimum temperature of the coldest month. Lines represent the 95th quantile of the density of points for each group. In each panel, symbols indicate some key vegetation types, with circular bullets designating remaining unlisted vegetation types.

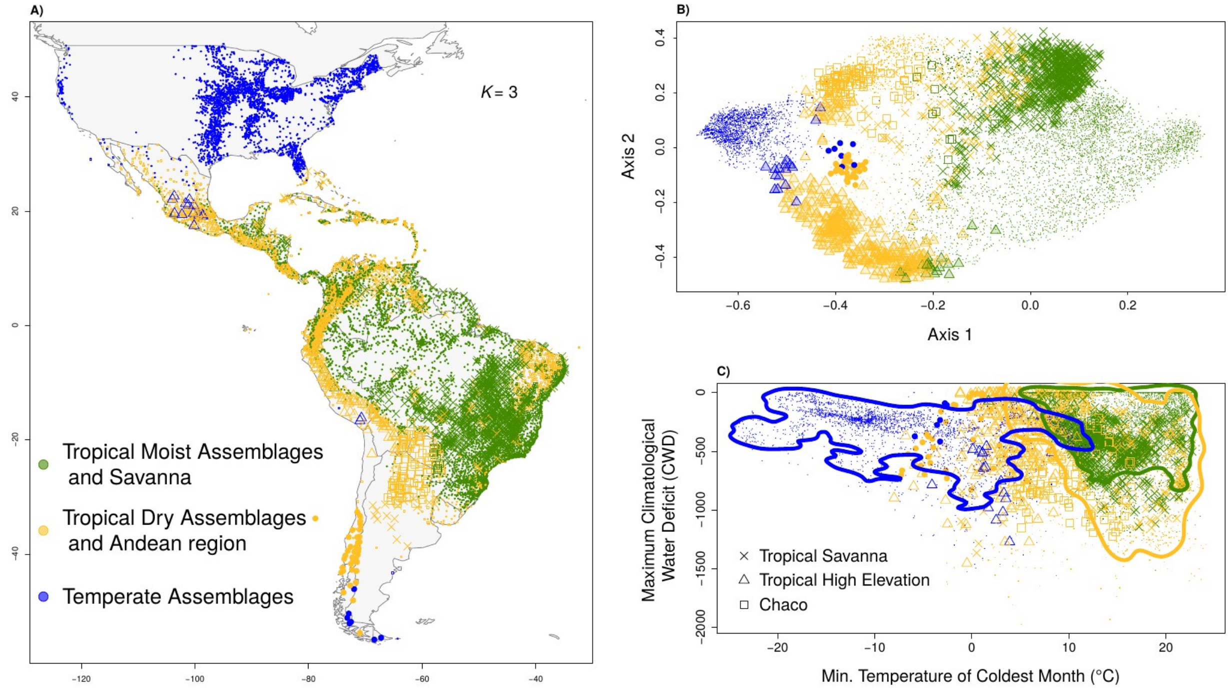

Fig. S5. Clustering *K* = 5. A) Location of the 9937 angiosperm tree assemblages in five evolutionary groups; B) Ordination of tree assemblages based on evolutionary lineage composition; C) Maximum Climatological Water Deficit (CWD) versus minimum temperature of the coldest month. Lines represent the 95th quantile of the density of points for each group. In each panel, symbols indicate some key vegetation types, with circular bullets designating remaining unlisted vegetation types.

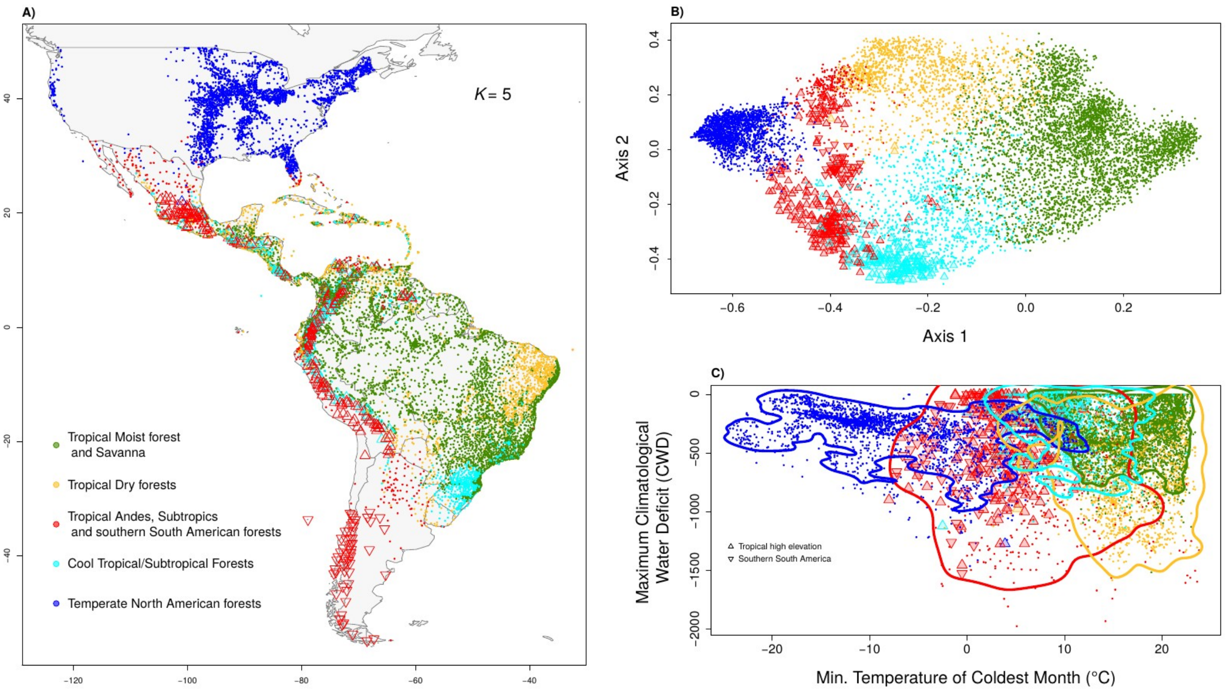

Fig. S6 Climatic organization of clusters in *K*=4. Density plots for sites assigned to Dry Tropical Group (yellow) vs. Wet Tropical Group (green) and northern extratropics (blue) vs. southern extratropics (red) across the environmental variables: Mean Annual Precipitation (MAP), Mean Annual Temperature (MAT), Temperature Seasonality (TS), and Maximum Climatic Water Deficit (CWD). Each plot show the Akaike Information Criterion (AIC) for four mixed models (logistic) using as a response variable the evolutionary grouping and as potential explanatory variables, each of the environmental variables in turn.

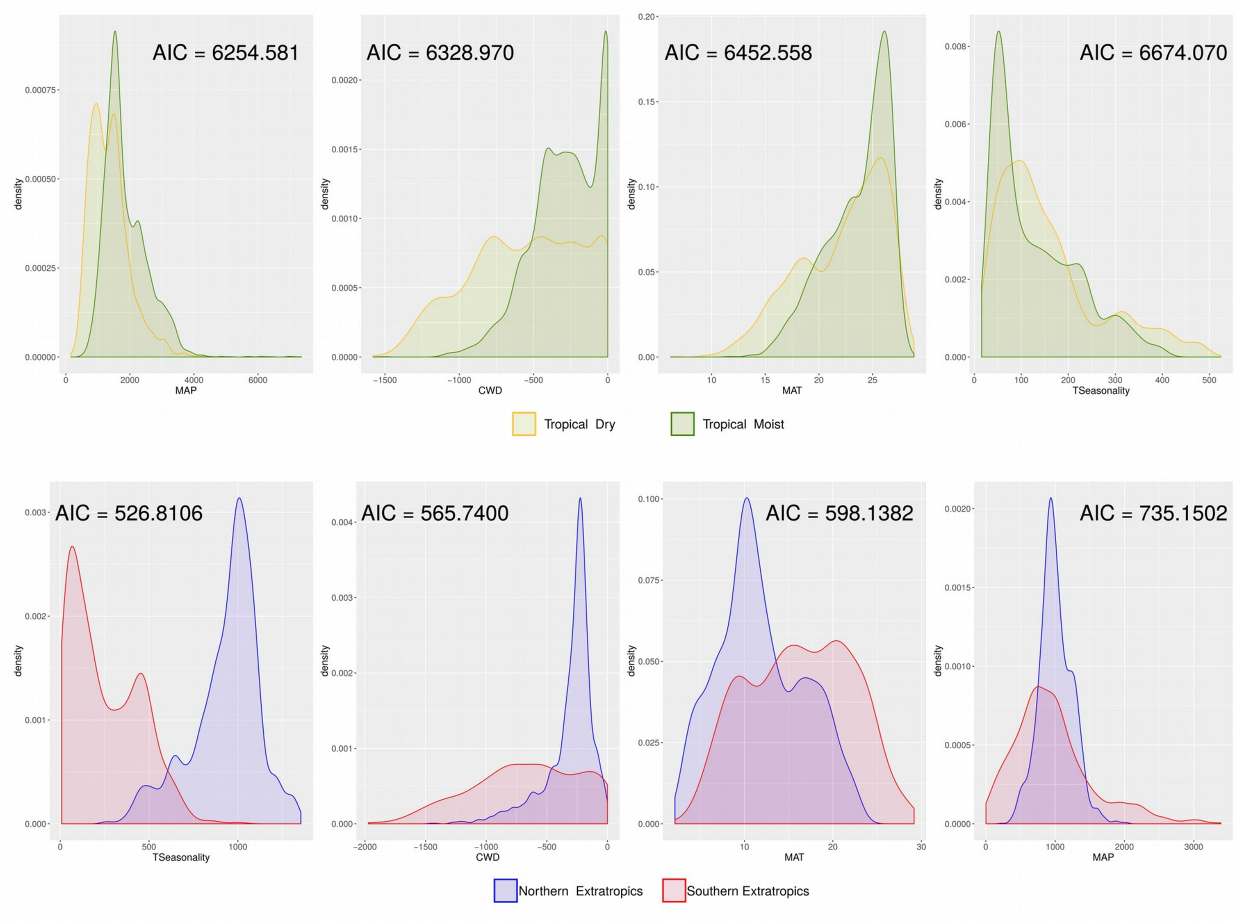

REFERENCES AND NOTES

1. A. R. Wallace, *The Geographical Distribution of Animals: With a Study of the Relations of Living and Extinct Faunas as Elucidating the Past Changes of the Earth's Surface* (Harper Brothers, 1876), vol. 1.

2. A. Takhtajan, *Floristic Regions of the World* (University of California Press, 1986), vol. 581.

3. B. G. Holt, J.-P. Lessard, M. K. Borregaard, S. A. Fritz, M. B. Araujo, D. Dimitrov, P.-H. Fabre, C. H. Graham, G. R. Graves, K. A. Jonsson, D. Nogues-Bravo, Z. Wang, R. J. Whittaker, J. Fjeldsa, C. Rahbek, An update of Wallace's zoogeographic regions of the world. *Science* **339**, 74–78 (2013).

4. R. Morley, Interplate dispersal paths for megathermal angiosperms. *Perspect. Plant Ecol. Syst.* **6**, 5–20 (2003).

5. R. T. Pennington, C. W. Dick, The role of immigrants in the assembly of the South American rainforest tree flora. *Philos. Trans. R Soc. Lond. B Biol. Sci.* **359**, 1611–1622 (2004).

6. S. Renner, Plant dispersal across the tropical atlantic by wind and sea currents. *Int. J. Plant. Sci.* **165**, S23–S33 (2004).

7. M. Lavin, B. P. Schrire, G. Lewis, R. T. Pennington, A. Delgado-Salinas, M. Thulin, C. E. Hughes, A. B. Matos, M. F. Wojciechowski, Metacommunity process rather than continental tectonic history better explains geographically structured phylogenies in legumes. *Philos. Trans. R Soc. Lond. B Biol. Sci.* **359**, 1509–1522 (2004).

8. J. F. Slik, J. Franklin, V. Arroyo-Rodríguez, R. Field, S. Aguilar, N. Aguirre, J. Ahumada, S.-I. Aiba, L. F. Alves, K. Anitha, A. Avella, F. Mora, G. A. C. Aymard, S. Báez, P. Balvanera, M. L. Bastian, J. F. Bastin, P. J. Bellingham, E. Van Den Berg, P. D. C. Bispo, P. Boeckx, K. Boehning-Gaese, F. Bongers, B. Boyle, F. Brambach, F. Q. Brearley, S. Brown, S. L. Chai, R. L. Chazdon, S. Chen, P. Chhang, G. Chuyong, C. Ewango, I. M. Coronado, J. Cristóbal-Azkarate, H. Culmsee, K. Damas, H. S. Dattaraja, P. Davidar, S. J. De Walt, H. DIn, D. R. Drake, A. Duque, G. Durigan, K. Eichhorn, E. S. Eler, T. Enoki, A. Ensslin, A. B. Fandohan, N. Farwig, K. J. Feeley, M. Fischer, O. Forshed, Q. S. Garcia, S. C. Garkoti, T. W. Gillespie, J. F. Gillet, C. Gonmadje, I. G. De La Cerda, D. M. Griffith, J. Grogan, K. R. Hakeem, D. J. Harris, R. D. Harrison, A. Hector, A. Hemp, J. Homeier, M. S. Hussain, G. Ibarra-Manríquez, I. F. Hanum, N. Imai, P. A. Jansen, C. A. Joly, S. Joseph, K. Kartawinata, E. Kearsley, D. L. Kelly, M. Kessler, T. J. Killeen, R. M. Kooyman, Y. Laumonier, S. G. Laurance, W. F. Laurance, M. J. Lawes, S. G. Letcher, J. Lindsell, J. Lovett, J. Lozada, X. Lu, A. M. Lykke, K. B. Mahmud, N. P. D. Mahayani, A. Mansor, A. R. Marshall, E. H. Martin, D. C. L. Matos, J. A. Meave, F. P. L. Melo, Z. H. A. Mendoza, F. Metali, V. P. Medjibe, J. P. Metzger, T. Metzker, D. Mohandass, M. A. Munguía-Rosas, R. Muñoz, E. Nurtjahy, E. L. De Oliveira, Onrizal, P. Parolin, M. Parren, N. Parthasarathy, E. Paudel, R. Perez, E. A. Pérez-García, U. Pommer, L. Poorter, L. Qi, M. T. F. Piedade, J. R. R. Pinto, A. D. Poulsen, J. R. Poulsen, J. S. Powers, R. C. Prasad, J. P. Puyravaud, O. Rangel, J. Reitsma, D. S. B. Rocha, S. Rolim, F. Rovero, A. Rozak, K. Ruokolainen, E. Rutishauser, G. Rutten, M. N. M. Said, F. Z. Saiter, P. Saner, B. Santos, J. R. D. Santos, S. K. Sarker, C. B. Schmitt, J. Schoengart, M. Schulze, D. Sheil, P. Sist, A. F. Souza, W. R.

Spironello, T. Sposito, R. Steinmetz, T. Stevart, M. S. Suganuma, R. Sukri, A. Sultana, R. Sukumar, T. Sunderland, H. S. S. Supriyadi, E. Suzuki, M. Tabarelli, J. Tang, E. V. J. Tanner, N. Targhetta, I. Theilade, D. Thomas, J. Timberlake, M. D. M. Valeriano, J. Van Valkenburg, T. Van Do, H. Van Sam, J. H. Vandermeer, H. Verbeeck, O. R. Vetaas, V. Adekunle, S. A. Vieira, C. O. Webb, E. L. Webb, T. Whitfeld, S. Wich, J. Williams, S. Wiser, F. Wittmann, X. Yang, C. Y. A. Yao, S. L. Yap, R. A. Zahawi, R. Zakaria, R. Zang, Phylogenetic classification of the world's tropical forests. *Proc. Natl. Acad. Sci. U.S.A.* **115**, 1837–1842 (2018).

9. M. D. Crisp, M. T. K. Arroyo, L. G. Cook, M. A. Gandolfo, G. J. Jordan, M. S. McGlone, P. H. Weston, M. Westoby, P. Wilf, H. P. Linder, Phylogenetic biome conservatism on a global scale. *Nature* **458**, 754–756 (2009).

10. E. Gagnon, J. J. Ringelberg, A. Bruneau, G. P. Lewis, C. E. Hughes, Global succulent biome phylogenetic conservatism across the pantropical Caesalpinia Group (Leguminosae). *New Phytol.* **222**, 1994–2008 (2018).

11. S. Pavoine, A guide through a family of phylogenetic dissimilarity measures among sites. *Oikos* **125**, 1719–1732 (2016).

12. R. T. Pennington, M. Lavin, A. Oliveira-Filho, Woody plant diversity, evolution, and ecology in the tropics: Perspectives from seasonally dry tropical forests. *Annu. Rev. Ecol. Evol. Syst.* **40**, 437–457 (2009).

13. L. Breiman, Random forests. *Mach. Learn.* **45**, 5–32 (2001).

14. P. Wardle, New Zealand timberlines. 1. Growth and survival of native and introduced tree species in the Craigieburn Range, Canterbury. *New Zeal. J. Bot.* **23**, 219–234 (1985).

15. V. Markgraf, M. McGlone, G. Hope, Neogene paleoenvironmental and paleoclimatic change in southern temperate ecosystems—A southern perspective. *Trends Ecol. Evol.* **10**, 143–147 (1995).

16. A. E. Zanne, D. C. Tank, W. K. Cornwell, J. M. Eastman, S. A. Smith, R. G. FitzJohn, D. J. McGlinn, B. C. O'Meara, A. T. Moles, P. B. Reich, D. L. Royer, D. E. Soltis, P. F. Stevens, M. Westoby, I. J. Wright, L. Aarssen, R. I. Bertin, A. Calaminus, R. Govaerts, F. Hemmings, M. R. Leishman, J. Oleksyn, P. S. Soltis, N. G. Swenson, L. Warman, J. M. Beaulieu, Three keys to the radiation of angiosperms into freezing environments. *Nature* **506**, 89–92 (2014).

17. C. Jaramillo, A. Cárdenas, Global warming and neotropical rainforests: A historical perspective. *Annu. Rev. Earth Planet. Sci.* **41**, 741–766 (2013).

18. P. E. Jardine, G. J. Harrington, J. A. Sessa, J. Daskova, Drivers and constraints on floral latitudinal diversification gradients. *J. Biogeogr.* **45**, 1408–1419 (2018).

19. A. J. Kerkhoff, P. E. Moriarty, M. D. Weiser, The latitudinal species richness gradient in New World woody angiosperms is consistent with the tropical conservatism hypothesis. *Proc. Natl. Acad. Sci. U.S.A.* **111**, 8125–8130 (2014).

20. T. Van der Hammen, J. Werner, H. Van Dommelen, Palynological record of the upheaval of the Northern Andes: A study of the Pliocene and lower quaternary of the Colombian Eastern Cordillera and the early evolution of its high-Andean biota. *Rev. Palaeobot. Palynol.* **16**, 1–122 (1973).

21. M. J. Donoghue, A phylogenetic perspective on the distribution of plant diversity. *Proc. Natl. Acad. Sci. U.S.A.* **105**, 11549–11555 (2008).

22. H. Hooghiemstra, Quaternary and upper-pliocene glaciations and forest development in the tropical andes: Evidence from a long high-resolution pollen record from the sedimentary basin of Bogotá, Colombia. *Palaeogeogr. Palaeoclimatol. Palaeoecol.* **72**, 11–26 (1989).

23. C. Hughes, R. Eastwood, Island radiation on a continental scale: Exceptional rates of plant diversification after uplift of the andes. *Proc. Natl. Acad. Sci. U.S.A.* **103**, 10334–10339 (2006).

24. B. R. Moore, M. J. Donoghue, Correlates of diversification in the plant clade dipsacales: Geographic movement and evolutionary innovations. *Am. Nat.* **170**, S28–S55 (2007).

25. C. D. Bell, A. Kutschker, M. T. Arroyo, Phylogeny and diversification of Valerianaceae (Dipsacales) in the southern Andes. *Mol. Phylogenet. Evol.* **63**, 724–737 (2012).

26. C. D. Bacon, F. J. Velásquez-Puentes, L. F. Hinojosa, T. Schwartz, B. Oxelman, B. Pfeil, M. T. Arroyo, L. Wanntorp, A. Antonelli, Evolutionary persistence in *Gunnera* and the contribution of southern plant groups to the tropical andes biodiversity hotspot. *PeerJ* **6**, e4388 (2018).

27. J. Wiens, M. Donoghue, Historical biogeography, ecology and species richness. *Trends Ecol. Evol.* **19**, 639–644 (2004).

28. E. N. H. Coronado, K. G. Dexter, R. T. Pennington, J. Chave, S. L. Lewis, M. N. Alexiades, E. Alvarez, A. A. de Oliveira, I. L. Amaral, A. Araujo-Murakami, E. J. M. M. Arets, G. A. Aymard, C. Baraloto, D. Bonal, R. Brienen, C. Ceron, F. C. Valverde, A. Di Fiore, W. Farfan-Rios, T. R. Feldpausch, N. Higuchi, I. Huamantupa-Chuquimaco, S. G. Laurance, W. F. Laurance, G. Lopez-Gonzalez, B. S. Marimon, B. H. Marimon-Junior, A. Monteagudo Mendoza, D. Neill, W. Palacios Cuenca, M. C. Penuela Mora, N. C. A. Pitman, A. Prieto, C. A. Quesada, H. Ramirez Angulo, A. Rudas, A. R. Ruschel, N. Salinas Revilla, R. P. Salomao, A. S. de Andrade, M. R. Silman, W. Spironello, H. ter Steege, J. Terborgh, M. Toledo, L. Valenzuela Gamarra, I. C. G. Vieira, E. Vilanova Torre, V. Vos, O. L. Phillips, Phylogenetic diversity of Amazonian tree communities. *Divers. Distrib*. **21**, 1295–1307 (2015).

29. A. Esquivel-Muelbert, T. R. Baker, K. G. Dexter, S. L. Lewis, H. ter Steege, G. Lopez Gonzalez, A. Monteagudo Mendoza, R. Brienen, T. R. Feldpausch, N. Pitman, A. Alonso, G. van der Heijden, M. Peña-Claros, M. Ahuite, M. Alexiaides, E. Á. Dávila, A. A. Murakami, L. Arroyo, M. Aulestia, H. Balslev, J. Barroso, R. Boot, A. Cano, V. C. Moscoso, J. A. Comiskey, F. Cornejo, F. Dallmeier, D. C. Daly, N. Dávila, J. F. Duivenvoorden, A. J. D. Montoya, T. Erwin, A. D. Fiore, T. Fredericksen, A. Fuentes, R. García-Villacorta, T. Gonzales, J.E. G. Andino, E. N. Honorio Coronado, I. Huamantupa-Chuquimaco, R. E. M. Jiménez, T. J. Killeen, Y. Malhi, C. Mendoza, H. Mogollón, P. M. Jørgensen, J. C. Montero, B. Mostacedo, W. Nauray, D. Neill, P. N. Vargas, S. Palacios, W. P.

Cuenca, N. C. P. Camacho, J. Peacock, J. F. Phillips, G. Pickavance, C. A. Quesada, H. Ramírez-Angulo, Z. Restrepo, C. R. Rodriguez, M. R. Paredes, M. C. Peñuela-Mora, R. Sierra, M. Silveira, P. Stevenson, J. Stropp, J. Terborgh, M. Tirado, M. Toledo, A. Torres-Lezama, M. N. Umaña, L. E. Urrego, R. V. Martinez, L. V. Gamarra, C. I. A. Vela, E. V. Torre, V. Vos, P. von Hildebrand, C. Vriesendorp, O. Wang, K. R. Young, C. E. Zartman, O. L. Phillips, Seasonal drought limits tree species across the neotropics. *Ecography* **40**, 618–629 (2017).

30. B. Schrire, M. Lavin, G. Lewis, in *Plant Diversity and Complexity Patterns: Local, Regional and Global Dimensions*, B. H. Friis, Ed. (Biologiske Skrifter, 2004), vol. 55, pp. 375–422.

31. M. F. Simon, R. Grether, L. P. de Queiroz, C. Skema, R. T. Pennington, C. E. Hughes, Recent assembly of the Cerrado, a neotropical plant diversity hotspot, by in situ evolution of adaptations to fire. *Proc. Natl. Acad. Sci. U.S.A.* **106**, 20359–20364 (2009).

32. R. T. Pennington, G. P. Lewis, J. A. Ratter, in *Neotropical Savannas and Seasonally Dry Forests* (CRC Press, 2006), pp. 17–45.

33. DRYFLOR, K. Banda-R, A. Delgado-Salinas, K. G. Dexter, R. Linares-Palomino, A. Oliveira-Filho, D. Prado, M. Pullan, C. Quintana, R. Riina, G. M. Rodríguez M., J. Weintritt, P. Acevedo-Rodríguez, J. Adarve, E. Álvarez, A. Aranguren B., J. C. Arteaga, G. Aymard, A. Castaño, N. Ceballos-Mago, Á. Cogollo, H. Cuadros, F. Delgado, W. Devia, H. Dueñas, L. Fajardo, Á. Fernández, M. Á. Fernández, J. Franklin, E. H. Freid, L. A. Galetti, R. Gonto, R. González-M., R. Graveson, E. H. Helmer, Á. Idárraga, R. López, H. Marcano-Vega, O. G. Martínez, H. M. Maturo, M. M. Donald, K. M. Laren, O. Melo, F. Mijares, V. Mogni, D. Molina, N. del Pilar Moreno, J. M. Nassar, D. M. Neves, L. J. Oakley, M. Oatham, A. R. Olvera-Luna, F. F. Pezzini, O. J. Reyes Dominguez, M. E. Ríos, O. Rivera, N. Rodríguez, A. Rojas, T. Särkinen, R. Sánchez, M. Smith, C. Vargas, B. Villanueva, R. T. Pennington, Plant diversity patterns in neotropical dry forests and their conservation implications. *Science* **353**, 1383–1387 (2016).

34. T. Kuemmerle, M. Altrichter, G. Baldi, M. Cabido, M. Camino, E. Cuellar, R. L. Cuellar, J. Decarre, S. Díaz, I. Gasparri, G. Gavier-Pizarro, R. Ginzburg, A. J. Giordano, H. R. Grau, E. Jobbágy, G. Leynaud, L. Macchi, M. Mastrangelo, S. D. Matteucci, A. Noss, J. Paruelo, M. Piquer-Rodríguez, A. Romero-Muñoz, A. Semper-Pascual, J. Thompson, S. Torrella, R. Torres, J. N. Volante, A. Yanosky, M. Zak, Forest conservation: Remember gran chaco. *Science* **355**, 465–465 (2017).

35. A. Oliveira-Filho, *NeoTropTree, Flora arbórea da Região Neotropical: Um banco de dados envolvendo biogeografia, diversidade e conservação*, Belo Horizonte: Universidade Federal de Minas Gerais (2017); http://www.neotroptree.info.

36. E. Burrill, A. Wilson, J. Turner, S. Pugh, J. Menlove, G. Christiansen, B. Conkling, W. David, *The Forest Inventory and Analysis Database: Database description and user guide version 8.0 for Phase 2. U.S.*, Department of Agriculture, Forest Service (2018); www.fia.fs.fed.us/library/database-documentation.

37. B. S. Maitner, B. Boyle, N. Casler, R. Condit, J. Donoghue II, S. M. Durán, D. Guaderrama, C. E. Hinchliff, P. M. Jørgensen, N. J. B. Kraft, B. M. Gill, C. Merow, N. Morueta-Holme, R. K. Peet, B.

Sandel, M. Schildhauer, S. A. Smith, J.-C. Svenning, B. Thiers, C. Violle, S. Wiser, B. J. Enquist, The BIEN R package: A tool to access the Botanical Information and Ecology Network (BIEN) database. *Methods Ecol. Evol.* **9**, 373–379 (2018).

38. P. L. Silva de Miranda, A. T. Oliveira-Filho, R. T. Pennington, D. M. Neves, T. R. Baker, K. G. Dexter, Using tree species inventories to map biomes and assess their climatic overlaps in lowland tropical South America. *Global Ecol. Biogeogr.* **27**, 899–912 (2018).

39. C. Baraloto, O. J. Hardy, C. T. Paine, K. G. Dexter, C. Cruaud, L. T. Dunning, M.-A. Gonzalez, J.-F. Molino, D. Sabatier, V. Savolainen, J. Chave, Using functional traits and phylogenetic trees to examine the assembly of tropical tree communities. *J. Ecol.* **100**, 690–701 (2012).

40. K. Dexter, J. Chave, Evolutionary patterns of range size, abundance and species richness in amazonian angiosperm trees. *PeerJ* **4**, e2402 (2016).

41. K. Katoh, D. M. Standley, MAFFT multiple sequence alignment software version 7: Improvements in performance and usability. *Mol. Biol. Evol.* **30**, 772–780 (2013).

42. A. Stamatakis, P. Hoover, J. Rougemont, A rapid bootstrap algorithm for the RAxML web servers. *Syst. Biol.* **57**, 758–771 (2008).

43. M. Gastauer, M. Neto, J. A. Alves, Updated angiosperm family tree for analyzing phylogenetic diversity and community structure. *Acta Bot. Bras.* **31**, 191–198 (2017).

44. S. A. Smith, B. C. O'Meara, treePL: Divergence time estimation using penalized likelihood for large phylogenies. *Bioinformatics* **28**, 2689–2690 (2012).

45. S. Magallón, S. Gómez-Acevedo, L. L. Sánchez-Reyes, T. Hernández-Hernández, A metacalibrated time-tree documents the early rise of flowering plant phylogenetic diversity. *New Phytol.* **207**, 437–453 (2015).

46. J. A. Bryant, C. Lamanna, H. Morlon, A. J. Kerkhoff, B. J. Enquist, J. L. Green, Microbes on mountainsides: Contrasting elevational patterns of bacterial and plant diversity. *Proc. Natl. Acad. Sci. U.S.A.* **105**, 11505–11511 (2008).

47. C. Tsirogiannis, B. Sandel, Phylomeasures: A package for computing phylogenetic biodiversity measures and their statistical moments. *Ecography* **39**, 709–714 (2016).

48. M. Maechler, P. Rousseeuw, A. Struyf, M. Hubert, K. Hornik, *cluster: Cluster Analysis Basics and Extensions* (2019). R package version 2.1.0—For new features, see the 'Changelog' file (in the package source).

49. S. Pavoine, adiv: Analysis of Diversity (2018) R package version 1.2.

50. K. J. Feeley, J. T. Stroud, Where on Earth are the "tropics"? *Front. Biogeogr.* **10**, 1–7 (2018).

51. R. J. Hijmans, S. E. Cameron, J. L. Parra, P. G. Jones, A. Jarvis, Very high resolution interpolated climate surfaces for global land areas. *Int. J. Climatol.* **25**, 1965–1978 (2005).

52. J. Chave, M. Réjou-Méchain, A. Búrquez, E. Chidumayo, M. S. Colgan, W. B. Delitti, A. Duque, T. Eid, P. M. Fearnside, R. C. Goodman, M. Henry, A. Martínez-Yrízar, W. A. Mugasha, H. C. Muller-Landau, M. Mencuccini, B. W. Nelson, A. Ngomanda, E. M. Nogueira, E. Ortiz-Malavassi, R. Pélissier, P. Ploton, C. M. Ryan, J. G. Saldarriaga, G. Vieilledent, Improved allometric models to estimate the aboveground biomass of tropical trees. *Glob. Chang. Biol.* **20**, 3177–3190 (2014).

53. D. Bates, M. Mächler, B. Bolker, S. Walker, Fitting linear mixed-effects models using lme4. *J. Stat. Softw.* **67**, 1–48 (2015).

54. M. De Cáceres, P. Legendre, Associations between species and groups of sites: Indices and statistical inference. *Ecology* **90**, 3566–3574 (2009).

55. M. De Caceres, F. Jansen, *Indicspecies: Relationship Between Species and Groups of Sites* (R package, 2016).

56. J. Larsson, *Eulerr: Area-Proportional Euler and Venn Diagrams with Ellipses* (R package version 3.1, 2018).

57. A. Dusa, *Venn: Draw Venn Diagrams* (R package version 1.7, 2018).

58. M. L. Bueno, K. G. Dexter, R. T. Pennington, V. Pontara, D. M. Neves, J. A. Ratter, A. T. de Oliveira-Filho, The environmental triangle of the Cerrado Domain: Ecological factors driving shifts in tree species composition between forests and savannas. *J. Ecol.* **106**, 2109–2120 (2018).

59. K. E. Armstrong, G. N. Stone, J. A. Nicholls, E. Valderrama, A. A. Anderberg, J. Smedmark, L. Gautier, Y. Naciri, R. Milne, J. E. Richardson, Patterns of diversification amongst tropical regions compared: A case study in Sapotaceae. *Front. Genet.* **5**, 362 (2014).

60. F. A. Carvalho, S. S. Renner, in *Molecular Phylogeny, Biogeography and an E-Monograph of the Papaya Family (Caricaceae) as an Example of Taxonomy in the Electronic Age* (Springer, 2015), pp. 49–81.

61. M. S. Appelhans, P. J. A. Keßler, E. Smets, S. G. Razafimandimbison, S. B. Janssens, Age and historical biogeography of the pantropically distributed Spathelioideae (Rutaceae, Sapindales). *J. Biogeogr.* **39**, 1235–1250 (2012).

62. T. R. Baker, R. T. Pennington, S. Magallon, E. Gloor, W. F. Laurance, M. Alexiades, E. Alvarez, A. Araujo, E. J. M. M. Arets, G. Aymard, A. A. de Oliveira, I. Amaral, L. Arroyo, D. Bonal, R. J. W. Brienen, J. Chave, K. G. Dexter, A. D. Fiore, E. Eler, T. R. Feldpausch, L. Ferreira, G. Lopez-Gonzalez, G. van der Heijden, N. Higuchi, E. Honorio, I. Huamantupa, T. J. Killeen, S. Laurance, C. Leaño, S. L. Lewis, Y. Malhi, B. S. Marimon, B. H. M. Junior, A. M. Mendoza, D. Neill, M. C. Peñuela-Mora, N. Pitman, A. Prieto, C. A. Quesada, F. Ramírez, H. R. Angulo, A. Rudas, A. R. Ruschel, R. P. Salomão, A. S. de Andrade, J. N. M. Silva, M. Silveira, M. F. Simon, W. Spironello,

H. ter Steege, J. Terborgh, M. Toledo, A. Torres-Lezama, R. Vasquez, I. C. G. Vieira, E. Vilanova, V. A. Vos, O. L. Phillips, Fast demographic traits promote high diversification rates of Amazonian trees. *Ecol. Lett.* **17**, 527–536 (2014).

63. C. D. Bell, M. J. Donoghue, Dating the Dipsacales: Comparing models, genes, and evolutionary implications. *Am. J. Bot*. **92**, 284–296 (2005).

64. L. Bardon, C. Sothers, G. T. Prance, P.-J. G. Malé, Z. Xi, C. C. Davis, J. Murienne, R. García-Villacorta, E. Coissac, S. Lavergne, J. Chave, Unraveling the biogeographical history of Chrysobalanaceae from plastid genomes. *Am. J. Bot*. **103**, 1089–1102 (2016).

65. A. Bruneau, M. Mercure, G. P. Lewis, P. S. Herendeen, Phylogenetic patterns and diversification in the Caesalpinioid legumes. *Botany* **86**, 697–718 (2008).

66. L. Cai, Z. Xi, K. Peterson, C. Rushworth, J. Beaulieu, C. C. Davis, Phylogeny of Elatinaceae and the tropical Gondwanan origin of the Centroplacaceae (Malpighiaceae, Elatinaceae) clade. *PLOS ONE* **11**, e0161881 (2016).

67. S. A. Catalano, J. C. Vilardi, D. Tosto, B. O. Saidman, Molecular phylogeny and diversification history of *Prosopis* (Fabaceae: Mimosoideae). *Biol. J. Linn. Soc.* **93**, 621–640 (2008).

68. S.-W. Chin, J. Shaw, R. Haberle, J. Wen, D. Potter, Diversification of almonds, peaches, plums and cherries–molecular systematics and biogeographic history of *Prunus* (Rosaceae). *Mol. Phylogenet. Evol.* **76**, 34–48 (2014).

69. D. M. Crayn, M. Rossetto, D. J. Maynard, Molecular phylogeny and dating reveals an Oligo-Miocene radiation of dry-adapted shrubs (former Tremandraceae) from rainforest tree progenitors (Elaeocarpaceae) in Australia. *Am. J. Bot*. **93**, 1328–1342 (2006).

70. L. A. Eserman, G. P. Tiley, R. L. Jarret, J. H. Leebens-Mack, R. E. Miller, Phylogenetics and diversification of morning glories (tribe Ipomoeeae, Convolvulaceae) based on whole plastome sequences. *Am. J. Bot.* **101**, 92–103 (2014).

71. E. M. Gardner, P. Sarraf, E. W. Williams, N. J. C. Zerega, Phylogeny and biogeography of *Maclura* (Moraceae) and the origin of an anachronistic fruit. *Mol. Phylogenet. Evol.* **117**, 49–59 (2017).

72. A. J. Harris, Q.-Y. Xiang, D. T. Thomas, Phylogeny, origin, and biogeographic history of *Aesculus* L.(Sapindales)—An update from combined analysis of DNA sequences, morphology, and fossils. *Taxon* **58**, 108–126 (2009).

73. J. Heckenhauer, R. Samuel, P. S. Ashton, B. Turner, M. H. J. Barfuss, T.-S. Jang, E. M. Temsch, J. Mccann, K. A. Salim, A. M. A. S. Attanayake, M. W. Chase, Phylogenetic analyses of plastid DNA suggest a different interpretation of morphological evolution than those used as the basis for previous classifications of Dipterocarpaceae (Malvales). *Bot. J. Linn. Soc.* **185**, 1–26 (2017).

74. L. Li, J. Li, J. G. Rohwer, H. van der Werff, Z.-H. Wang, H.-W. Li, Molecular phylogenetic analysis of the *Persea* group (Lauraceae) and its biogeographic implications on the evolution of tropical and subtropical Amphi-Pacific disjunctions. *Am. J. Bot.* **98**, 1520–1536 (2011).

75. A. R. Mast, E. F. Milton, E. H. Jones, R. M. Barker, W. R. Barker, P. H. Weston, Time calibrated phylogeny of the woody Australian genus *Hakea* (Proteaceae) supports multiple origins of insect-pollination among bird-pollinated ancestors. *Am. J. Bot.* **99**, 472–487 (2012).

76. I. Michalak, L.-B. Zhang, S. S. Renner, Trans-Atlantic, trans-Pacific and trans-Indian Ocean dispersal in the small Gondwanan Laurales family Hernandiaceae. *J. Biogeogr.* **37**, 1214–1226 (2010).

77. M. L. Milner, P. H. Weston, M. Rossetto, M. D. Crisp, Biogeography of the Gondwanan genus *Lomatia* (Proteaceae): Vicariance at continental and intercontinental scales. *J. Biogeogr.* **42**, 2440–2451 (2015).

78. L. C. Moyle, M. S. Olson, P. Tiffin, Patterns of reproductive isolation in three Angiosperm genera. *Evolution* **58**, 1195–1208 (2004).

79. A. N. Muellner, T. D. Pennington, A. V. Koecke, S. S. Renner, Biogeography of *Cedrela* (Meliaceae, Sapindales) in central and South America. *Am. J. Bot.* 97, 511–518 (2010).

80. S. S. Renner, L.-B. Zhang, J. Murata, A chloroplast phylogeny of *Arisaema* (Araceae) illustrates Tertiary floristic links between Asia, North America, and East Africa. *Am. J. Bot.* **91**, 881–888 (2004).

81. S. S. Renner, G. W. Grimm, G. M. Schneeweiss, T. F. Stuessy, R. E. Ricklefs, Rooting and dating maples (*Acer*) with an uncorrelated-rates molecular clock: Implications for North American/Asian disjunctions. *Syst. Biol.* **57**, 795–808 (2008).

82. S. S. Renner, J. S. Strijk, D. Strasberg, C. Thébaud, Biogeography of the Monimiaceae (Laurales): A role for East Gondwana and long-distance dispersal, but not West Gondwana. *J. Biogeogr.* **37**, 1227–1238 (2010).

83. A. Rockinger, A. S. Flores, S. S. Renner, Clock-dated phylogeny for 48% of the 700 species of *Crotalaria* (Fabaceae–Papilionoideae) resolves sections worldwide and implies conserved flower and leaf traits throughout its pantropical range. *BMC Evol. Biol.* **17**, 61 (2017).

84. J. G. Rodrigues, J. A. Lombardi, M. B. Lovato, Phylogeny of *Cissus* (Vitaceae) focusing on South American species. *Taxon* **63**, 287–298 (2014).

85. H. Sauquet, P. H. Weston, C. L. Anderson, N. P. Barker, D. J. Cantrill, A. R. Mast, V. Savolainen, Contrasted patterns of hyperdiversification in Mediterranean hotspots. *Proc. Natl. Acad. Sci. U.S.A.* **106**, 221–225 (2009).

86. J. V. Schneider, G. Zizka, Phylogeny, taxonomy and biogeography of Neotropical *Quiinoideae* (Ochnaceae s.l). *Taxon* **66**, 855–867 (2017).

87. O. Schwery, R. E. Onstein, Y. Bouchenak-Khelladi, Y. Xing, R. J. Carter, H. P. Linder, As old as the mountains: The radiations of the Ericaceae. *New Phytol.* **207**, 355–367 (2015).

88. J. E. E. Smedmark, A. A. Anderberg, Boreotropical migration explains hybridization between geographically distant lineages in the pantropical clade *Sideroxyleae* (Sapotaceae). *Am. J. Bot.* **94**, 1491–1505 (2007).

89. J. E. E. Smedmark, T. Eriksson, B. Bremer, Divergence time uncertainty and historical bio geography reconstruction–an example from *Urophylleae* (Rubiaceae). *J. Biogeogr.* **37**, 2260–2274 (2010).

90. M. H. Terra-Araujo, A. D. de Faria, A. Vicentini, S. Nylinder, U. Swenson, Species tree phylogeny and biogeography of the Neotropical genus *Pradosia* (Sapotaceae, Chrysophylloideae). *Mol. Phylogenet. Evol.* **87**, 1–13 (2015).

91. A. H. Thornhill, S. Y. W. Ho, C. Külheim, M. D. Crisp, Interpreting the modern distribution of Myrtaceae using a dated molecular phylogeny. *Mol. Phylogenet. Evol.* **93**, 29–43 (2015).

92. N. Thomas, J. J. Bruhl, A. Ford, P. H. Weston, Molecular dating of Winteraceae reveals a complex biogeographical history involving both ancient Gondwanan vicariance and long distance dispersal. *J. Biogeogr.* **41**, 894–904 (2014).

93. E. A. Tripp, L. A. McDade, A rich fossil record yields calibrated phylogeny for Acanthaceae (Lamiales) and evidence for marked biases in timing and directionality of intercontinental disjunctions. *Syst. Biol.* **63**, 660–684 (2014).

94. P. C. van Welzen, K. Pruesapan, I. R. H. Telford, J. J. Bruhl, Historical biogeography of *Breynia* (Phyllanthaceae): What caused speciation? *J. Biogeogr.* **42**, 1493–1502 (2015).

95. T. N. C. Vasconcelos, C. E. B. Proença, B. Ahmad, D. S. Aguilar, R. Aguilar, B. S. Amorim, K. Campbell, I. R. Costa, P. S. De-Carvalho, J. E. Q. Faria, A. Giaretta, P. W. Kooij, D. F. Lima, F. F. Mazine, B. Peguero, G. Prenner, M. F. Santos, J. Soewarto, E. J. Lucas, Myrteae phylogeny, calibration, biogeography and diversification patterns: Increased understanding in the most species rich tribe of Myrtaceae. *Mol. Phylogenet. Evol.* **109**, 113–137 (2017).

96. Q.-Y. J. Xiang, D. T. Thomas, Q. P. Xiang, Resolving and dating the phylogeny of Cornales—Effects of taxon sampling, data partitions, and fossil calibrations. *Mol. Phylogenet. Evol.* **59**, 123–138 (2011).

97. M.-Q. Yang, D.-Z. Li, J. Wen, T.-S. Yi, Phylogeny and biogeography of the amphi-pacific genus *Aphananthe*. *PLOS ONE* **12**, e0171405 (2017).

98. T. Yang, L.-M. Lu, W. Wang, J.-H. Li, S. R. Manchester, J. Wen, Z.-D. Chen, Boreotropical range expansion and long-distance dispersal explain two amphi-pacific tropical disjunctions in Sabiaceae. *Mol. Phylogenet. Evol.* **124**, 181–191 (2018).
